# Supplementary material for: Radiation-induced rescue effect on human breast carcinoma cells is regulated by macrophages
Source: Biochem Biophys Rep. 2025 Feb 8;41:101936. doi: 10.1016/j.bbrep.2025.101936 (PMC11850746; doi:10.1016/j.bbrep.2025.101936)
Supplement: Multimedia component 1 [file mmc1.pdf]

Authorship change request

Important information. Please read before completing this form.
This form is to request any change in authorship (additions, removals, or reordering) of a submitted manuscript, including changes in corresponding authors. This form should not be used for changes requested *after* publication or for name changes or corrections.

Prior to completing this form, all authors should carefully review the 'duties of authors' section of the Elsevier publishing ethics policy, and in particular, the sections on:

- Authorship of the paper
- The use of generative AI and AI-assisted technologies in scientific writing

Please also carefully review the journal's guide for authors (this might also be referred to as 'instructions for authors') as some journals may have additional authorship criteria (e.g., the ICMJE guidelines for authorship).

The publisher and editor cannot investigate or mediate any authorship disputes. If you are unable to obtain agreement from all authors, including those you intend to remove, we recommend seeking guidance from your institution. We will not consider your change request and will not proceed with the publication of your manuscript until all outstanding authorship disputes are resolved.

In order for the request to be considered, this completed form should be submitted in Editorial Manager with the cover letter for your revision.

Section 1. Submission information

To be completed by the corresponding author.

Submission information	
**Journal title**	Biochemistry and Biophysics Reports
**Manuscript number**	BBREP-D-24-00795
**Manuscript title**	Radiation-induced rescue effect on human breast carcinoma cells is regulated by macrophages

**Change(s) requested** (indicate as appropriate)	
☑ Add new author(s)	☐ Remove author(s)
☐ Change the corresponding author	☐ Change the order of authors

Section 2. Author(s) added or removed

Complete one table for each author to be added or removed. Please include as much detail as possible in the "Reason for change" section so that we can evaluate if the change is necessary. At a minimum, this should include an explanation for why the change is being requested and why the author was/was not included in the original author list.

If the form is incomplete, or the reasons provided are insufficiently detailed or do not address the points above, your request will be denied.

2.1 Author information

**Given/first name(s)**	Clement Manohar
**Family/last name**	Arava
**Email address**	clementmanohar7@gmail.com
**Institution**	Université de Rouen Normandie, Rouen, France
**Change(s) requested** (indicate as appropriate)	☑ Add new author ☐ Remove author ☐ Make the corresponding author

Individual contributions per CRediT Contributor Roles Taxonomy (complete for author additions only)

☐ Conceptualization	☐ Data curation	☐ Formal analysis
☐ Funding acquisition	☐ Investigation	☑ Methodology
☐ Project administration	☐ Resources	☐ Software
☐ Supervision	☐ Validation	☐ Visualization
☐ Writing – original draft	☑ Writing – review & editing	

Reason for the change

The new co-author, Clement Manohar Arava, a post-doctoral scholar specialized in biophysics and biomaterial sciences, contributed significantly in helping us address the journal reviewers' comments by assisting with the UV photon calculations. Additionally, he provided valuable feedback on the revised manuscript, offering insightful comments that greatly enhanced its clarity and quality during the editing process.

2.2 Author information

Given/first name(s)

Family/last name

Email address

Institution

Change(s) requested (indicate as appropriate) ☐ Add new author ☐ Remove author ☐ Make the corresponding author

Individual contributions [per CRediT Contributor Roles Taxonomy] (complete for author additions only)

☐ Conceptualization	☐ Data curation	☐ Formal analysis
☐ Funding acquisition	☐ Investigation	☐ Methodology
☐ Project administration	☐ Resources	☐ Software
☐ Supervision	☐ Validation	☐ Visualization
☐ Writing – original draft	☐ Writing – review & editing	

Reason for the change

2.3 Author information

Given/first name(s)

Family/last name

Email address

Institution

Change(s) requested (indicate as appropriate) ☐ Add new author ☐ Remove author ☐ Make the corresponding author

Individual contributions [per CRediT Contributor Roles Taxonomy] (complete for author additions only)

☐ Conceptualization	☐ Data curation	☐ Formal analysis
☐ Funding acquisition	☐ Investigation	☐ Methodology
☐ Project administration	☐ Resources	☐ Software
☐ Supervision	☐ Validation	☐ Visualization
☐ Writing – original draft	☐ Writing – review & editing	

Reason for the change

2.4 Author information

**Given/first name(s)**	
**Family/last name**	
**Email address**	
**Institution**	
**Change(s) requested** (indicate as appropriate)	☐ Add new author ☐ Remove author ☐ Make the corresponding author

Individual contributions per CRediT Contributor Roles Taxonomy (complete for author additions only)

☐ Conceptualization	☐ Data curation	☐ Formal analysis
☐ Funding acquisition	☐ Investigation	☐ Methodology
☐ Project administration	☐ Resources	☐ Software
☐ Supervision	☐ Validation	☐ Visualization
☐ Writing – original draft	☐ Writing – review & editing	

Reason for the change

2.5 Author information

**Given/first name(s)**	
**Family/last name**	
**Email address**	
**Institution**	
**Change(s) requested** (indicate as appropriate)	☐ Add new author ☐ Remove author ☐ Make the corresponding author

Individual contributions per CRediT Contributor Roles Taxonomy (complete for author additions only)

☐ Conceptualization	☐ Data curation	☐ Formal analysis
☐ Funding acquisition	☐ Investigation	☐ Methodology
☐ Project administration	☐ Resources	☐ Software
☐ Supervision	☐ Validation	☐ Visualization
☐ Writing – original draft	☐ Writing – review & editing	

Reason for the change

*Add additional page(s) as needed for more requested changes.

Section 3. Author order and agreement

Provide the author list in the order that you would like it to be published.

The form must be signed individually by each author, including any added/removed authors. In cases of multi-author collaborative or consortia groups the corresponding author must sign on behalf of the group.

While manual signatures are acceptable, we highly encourage the use of electronic signature software (DocuSign, Adobe Sign, HelloSign, or similar) with valid e-signatures. These signatures should reflect your institutional information and email, as provided in the author list below. Typed signatures or images of signatures will not be accepted.

By signing this form all authors agree:

1) that they have read and acknowledge the publishing ethics policies linked in the "Important Information" section of this form;
2) agree to the addition and/or removal of the authors listed in section 2 and to the revised order of the author list in this section 3, and;
3) that all information provided accurately reflects the authorship of the article.

Agreement of removed author(s)

Full name	Email address	Signature	Date

Proposed author list

Order	Full name	Email address	Signature	Date
01	Spoorthy Pathikonda	spoorthy.709@gmail.com	Spoorthy.P.S.	19 Dec 2024
02	Li Tian	litian5-c@my.cityu.edu.hk	Tian Li	23 Dec 2024
03	Clement Manohar Arava	clementmanohar7@gmail.com	[illegible]	20 Dec 2024
04	Shuk Han Cheng	bhcheng@cityu.edu.hk	[illegible]	23 Dec 2024
05	Yun Wah Lam	Y.Lam@hud.ac.uk	[illegible]	23/12/2024
06				
07				
08				
09				
10				

Proposed author list

Order	Full name	Email address	Signature	Date
11				
12				
13				
14				
15				
16				
17				
18				
19				
20				
21				
22				
23				
24				
25				
26				
27				
28				
29				
30				
31				
32				
33				
34				
35				

*Add additional page(s) as needed.
